# Understanding and optimizing fish counting techniques based on electrical impedance measurements

**DOI:** 10.1371/journal.pone.0293699

**Published:** 2023-10-30

**Authors:** Lukasz J. Nowak, Martin Lankheet

**Affiliations:** Wageningen University and Research, Wageningen, The Netherlands; Vellore Institute of Technology, INDIA

## Abstract

Sustainable aquatic resources management requires reliable methods for fish detection in various environmental conditions. Herein, we study fundamental mechanisms underlying the application of electrical impedance measurements in this regard. We present results of experimental studies conducted in laboratory conditions using a low-cost impedance measurement circuit, as well as the corresponding numerical models. We also present evaluation results of a newly developed, real-time detection algorithm based on adaptive thresholding. The numerical model was validated by extracting fish tracks in 3D space from the experimental datasets, and then comparing the calculated versus measured impedance values as functions of fish coordinates in time. Numerical predictions closely resemble the experimental data. The detection sensitivity and specificity values determined for various settings exceeded 90%. Electrode width to spacing ratio is demonstrated to be a crucial parameter influencing the system sensitivity distribution. The introduced approach can constitute a framework for designing electrical impedance-based fish counting systems.

## Introduction

### Background

Fish counting plays a crucial role in aquaculture, aquatic resources management, and estimating population abundances of various species. Fish monitoring techniques vary depending on a target environment and conditions. In general, they can be divided into two groups: methods based on fish sampling, or methods based on remote sensing. Here, we focus on remote sensing techniques, which have the advantage of being harmless to the animals and can be fully automated. In open water reservoirs acoustic imaging systems are an effective approach, due to the relatively low attenuation of acoustic waves in water, long imaging distances, and high achievable imaging resolution, independent of water optical transparency [1, 2]. Those benefits, however, are of less importance in confined spaces, such as various types of fish passages. In these cases the required detection distances are relatively short, and the presence of walls, water surface ripples, and air bubbles introduce multiple reflections of the ultrasound pulses and distortions to the received signal. Thus, other techniques are commonly employed for counting fish under such conditions.

If the water depth does not significantly exceed the visibility limits, passing fish can be counted based on visual observations. In the most direct approach this can be done manually, by trained observers located on shore. Such programs are commonly employed for monitoring fish populations in rivers. They have, however, several important disadvantages–including involvement of significant human resources, problems with standardization, and varying counting efficiency, depending on conditions [3]. For this reason many different approaches to replace human-based observations with automated fish counting techniques have been developed and implemented over the past decades, and remain the focus of continuing efforts [4].

Automatization of visual observations can be done by replacing human observers with cameras and employing image processing algorithms for extracting fish from captured video recordings. The cameras can also be installed underwater, which expands their potential applicability to areas inaccessible for human observers–such as, e.g., direct monitoring of fishing nets [5]. The reported fish detection accuracy based on image analysis varies from above 60% to over 90% [4]. The main challenges related to camera-based counting techniques are varying, and often poor visibility conditions underwater and relatively high required computational power for data extraction from the video stream.

Yet another approach to remote fish detection underwater is to measure electrical impedance using a set of submerged electrodes. This technique utilizes the fact that electrical properties of fish tissues differ from the corresponding characteristics of ambient water, and thus fish appearance in the vicinity of the electrodes manifests itself by observable changes in the determined impedance values. A description of a fish detection device utilizing this principle was published already in 1975 [6], with further reference to earlier investigations on automatic, electronic fish counters conducted by the US Navy in 1965. Commercial fish counters developed later on utilized only the real part of the complex impedance value–electrical resistance–for fish detection and are referred to in the literature as “resistivity counters”. Numerous studies reported performance evaluations of such commercial systems installed at various rivers, comparing resistivity counter data with outcomes of visual observations. Reddin et al. [7] reported perfect agreement between Atlantic salmon counts during a 26 day long test conducted in a Canadian river. Appleby and Tipping [8] tested counting efficiency of a commercial resistivity counter for different fish species stocked at rearing ponds, comparing the readings to results of hand counts. They found the mismatch to be within 1,4% to 7%, depending on the counting rate. Other studies reported significantly worse efficiency of commercial resistivity counters, indicating also possible sources of errors. Dunkley and Shearer [9] showed that smaller fish had a much higher chance of remaining undetected, compared to larger individuals. They also indicated that the sensitivity of the counter depended on electrical conductivity of the water. McCubbing et al. [10] reported counting efficiency of over 80%, except from the periods of saltwater intrusions caused by high tides. Forbes et al. [11] in their work refer to numerous other studies showing fish counting accuracy from below 60% to above 90%. Their own results indicated a slight overcount for fish above a threshold length of 50 cm, but no counts of individuals below this limit—with over 350 missed passes of such smaller fish identified on the corresponding video recordings. Sheppard and Bednarski [12] presented results of their evaluation of a resistivity counting system conducted over a course of 13 years. They showed a systematic underestimate of passing fish by 35–87%, indicating the need to improve the existing technology and increase the detection performance.

The results of the previously published studies on fish counting using resistance measurements indicate that this technique can potentially achieve high detection accuracy. However, it is also prone to failure under certain conditions, raising questions about the possible sources of errors and methods to mitigate their influence. These questions can only be answered by a thorough understanding of the mechanisms underlying the interaction of fish with low-intensity electric currents passed between a set of submerged electrodes. Previously published studies on fish enumeration using resistivity counters did not attempt to model or analyze the underlying principles governing electric fish counting, providing only observation results. They also all rely on different commercial fish counting devices, thus the available technical specifications of the system settings–and their reliability–are limited to information provided by the manufacturers. Various studies utilized significantly different electrode setups (flat panels with bar electrodes on the bottom, electrode fences in the stream cross-section, tunnels with ring electrodes, etc.) and operated in different conditions. This makes it difficult to draw any definitive conclusions based on the available aggregated data.

At the same time these studies showed that electrical impedance measurements have great potential for reliably counting fish, which make the technology worth further investigations and optimization. The sensor elements used–electrodes made of electrically conductive material–are cheap and easily manufactured in a wide variety of shapes and sizes, which creates possibilities for easy adjustment and optimization to specific environmental conditions. The bandwidth of the generated data stream and the required computational processing power is much lower compared to the camera-based fish counting systems. Electronic devices for impedance measurements can be inexpensive and energy efficient, enabling easy multiplication of measurement channels and long operating times when battery powered.

The main challenges associated with fish counting using electrical impedance measurements are highly non-uniform spatial distribution of detection sensitivity, which complicates the geometrical design of the electrodes. Moreover, fluctuations of electrical properties of water, and often poor signal to noise ratio make automatic detection challenging. We partially addressed these issues in our previous study [13] in which we investigated fish detection using a set of in-plane patch electrodes, focusing on fundamental mechanisms associated with the detection process. Here we investigate the application of electrical impedance measurements for counting fish passes between parallel strip electrodes. For this purpose, we developed an adaptive detection algorithm that self-adjusts to variable noise levels and fluctuating electrical conductivity of water in real-time. We studied the relation between the algorithm settings and detection performance, as well as the influence of electrodes’ geometry on spatial sensitivity distributions. In order to research the effects of individual factors and to keep other parameters under control, we conducted our studies in an artificial water tank, under laboratory conditions. We constructed the experimental setup using low-cost electronic components, bringing the outcomes of the study closer to potential real-world applications. Finally, we developed a finite element model that numerically simulates experimental results for different electrode configurations, and that can be used to optimally design dedicated detection systems. Our aim is to provide reliable foundations–in terms of hardware design, algorithm development, data processing techniques–for design, construction, and parameter optimization of electrical impedance-based fish counters.

### Electrical impedance measurements

Electrical impedance is a quantity that describes the relation between a harmonic voltage signal applied between electrodes and the resulting electric current intensity:

$$Z(\omega )=\frac{U_{PU}e^{-i\omega t}}{I_{CC}e^{-i\omega t+\varphi }},$$ (1)

Where *ω* = 2*πf*, and *f* is the signal frequency. $i=\sqrt{-1},\varphi$ is a phase shift between voltage and current signals, *U_PU_* denotes the amplitude of voltage signal measured between voltage pick up (PU) electrodes, and *I_CC_* is the amplitude of the electric current intensity flow between current carrying (CC) electrodes. PU and CC might refer to the same electrodes–such a case would be referred to as two-electrode configuration. However, for measurements in water we separate them to mitigate the effects of ionic double layer formation and biases introduced by the interface impedance [14, 15]. The advantages of using a four-electrode setup were studied in detail in our previous work [13].

Electrical impedance is a complex quantity and thus can be described as a sum of real and imaginary parts:

Z=Re(Z)+iIm(Z), (2)

where the real part *Re*(*Z*) is called the resistance and defines the voltage to current intensity ratio if signals are in phase–also including a special case of direct current excitation, i.e., when *f* = 0. The imaginary part *Im*(*Z*) is referred to as the reactance and reflects capacitive or inductive behavior of the investigated system. In case of purely biological samples *Im*(*Z*) is always negative, reflecting capacitive properties of cells [14].

Electrical impedance measurements utilize only low-intensity current and are completely harmless to fish, even when in direct contact with the measurement electrodes [16, 17].

## Methods

### Experimental setup and data processing

**Fig 1:**
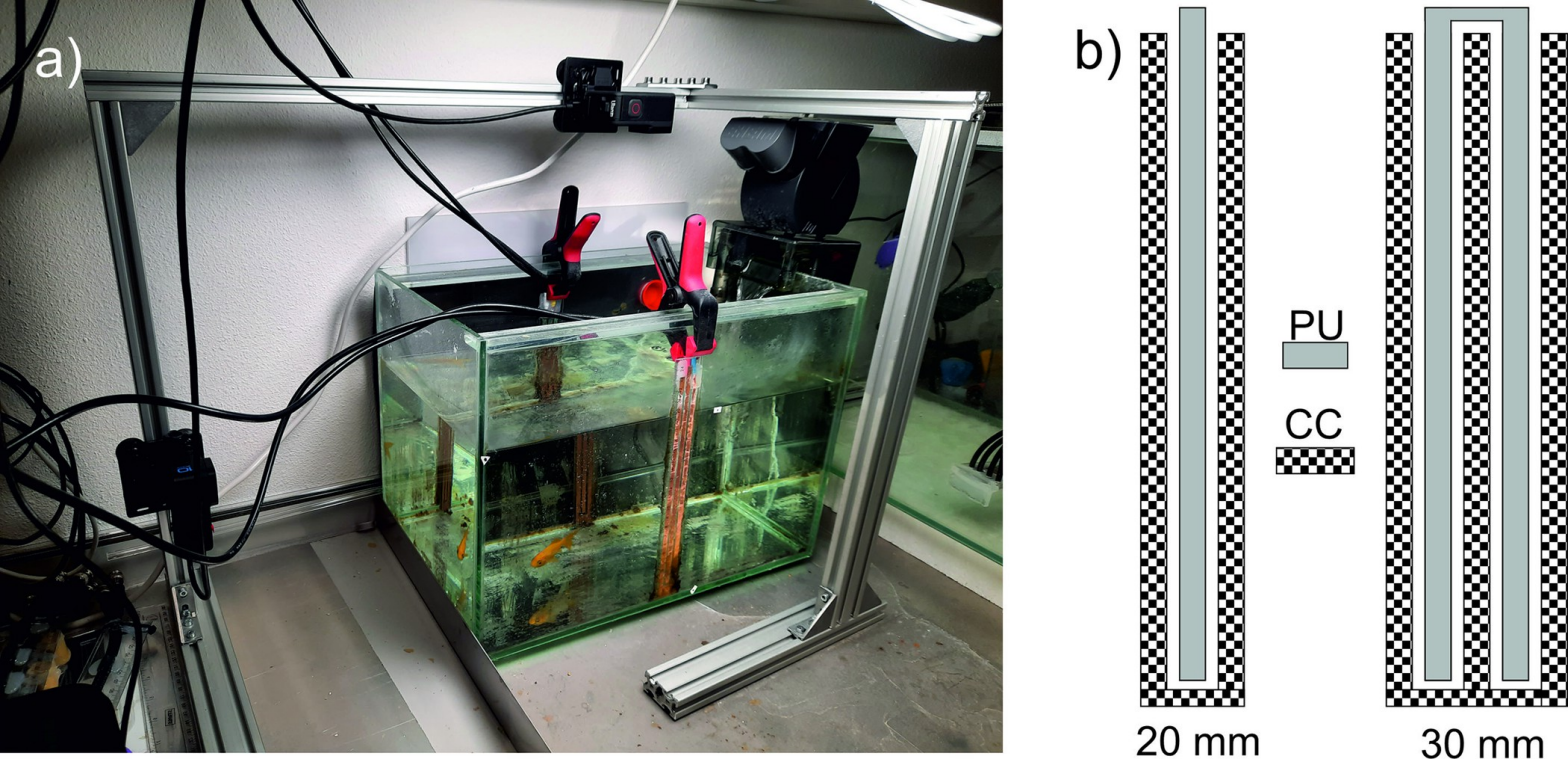
Laboratory setup used for experimental investigations. a) Fish tank with the measurement electrodes and two cameras for optical localization. b) The electrode design, each pair consisting of a current carrying (CC) and voltage pickup (PU) electrode.

The laboratory setup used for experimental investigations is presented in Fig 1. The setup consists of a glass water tank with inner dimensions 400 × 240 × 295 mm (x × y × z; length times width times height), filled with water to a level of 200 mm. Inside the tank a single goldfish (*Carassius auratus*) was swimming freely. The standard length of the fish was approximately 50 mm.

The measurement electrodes were made of strips of conductive copper tape and were located in the middle of the longer walls of the tank, facing each other. Separation of PU and CC electrodes was achieved by forming two interlocking, insulated comb-like shapes. Two different electrode sets were used, with total widths of 20 and 30 mm. The electrodes extended from the bottom of the tank to above the water level, and spacing between them was equal to the tank width, i.e., 240 mm.

The impedance measurements were conducted using a low-cost biomedical analog front-end (AFE) AD5940 (Analog Devices, USA). We used an evaluation board with ADICUP3029 microcontroller and customized in-house software for communication. The measurement device was connected to a host computer via a USB interface. The data was read from AFE at a rate of approximately 20 reads per second and streamed continuously through a serial port. Each data frame contained information about the device identification number, signal frequency, determined resistance and reactance values, and a time stamp. The signal frequency was set to 50 kHz, based on findings in previous experiments [13]. Data acquisition and processing by the host computer was conducted using Python scripts.

Due to the fact that the experiments conducted within the framework of the present study included only non-invasive measurements with the freely-swimming fish and that electrical impedance measurements do not cause any harm or stress to the animal [13, 16, 17] the ethical approval was in this case not required.

**Fig 2:**
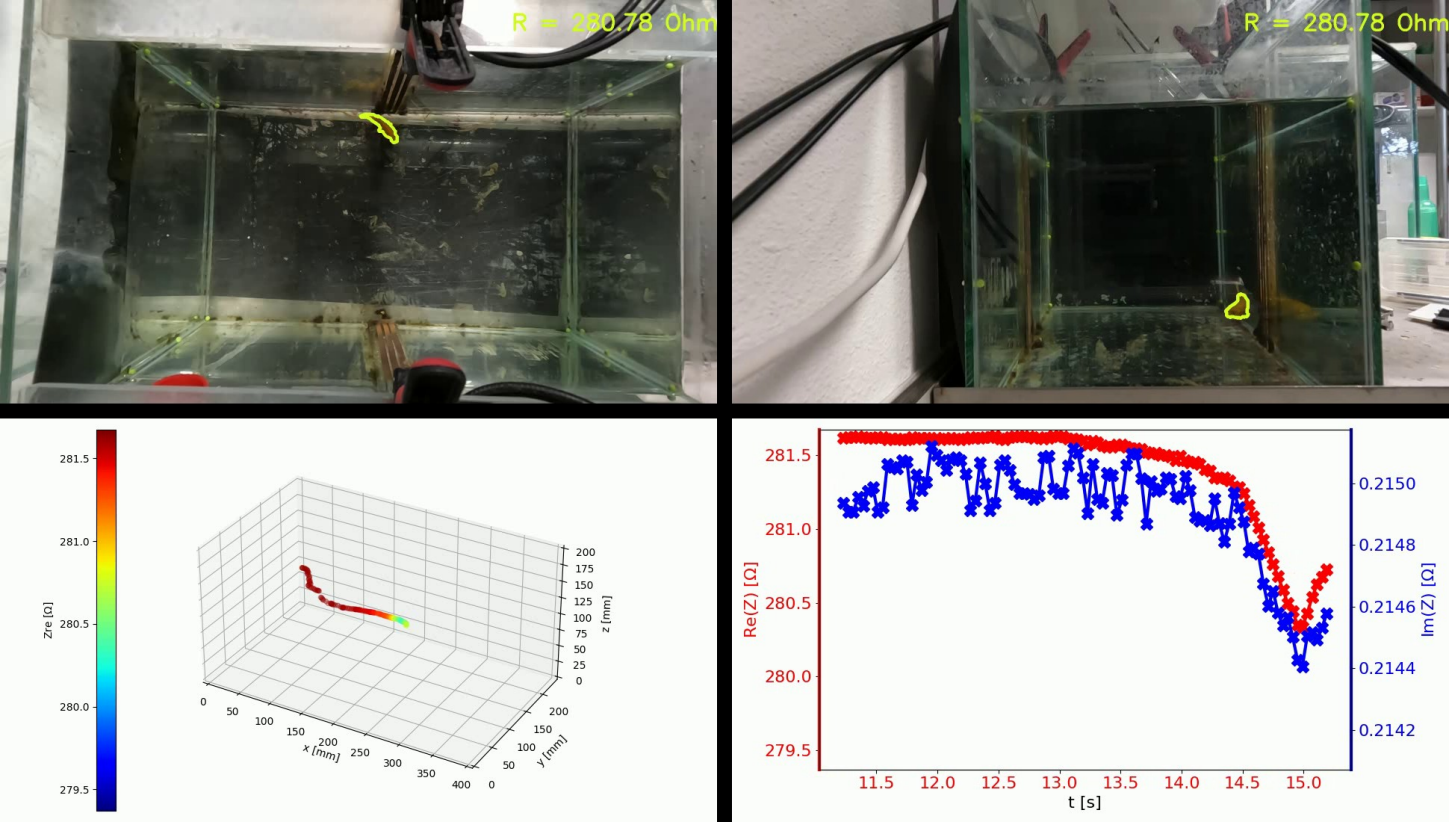
Optical fish localization with synchronized impedance measurements. Camera views with determined fish contours (top), reconstructed fish track in 3D space (bottom left), and synchronized waveform plots of real and imaginary impedance parts (bottom right).

Fish location inside the tank was continuously monitored using two GoPro Hero 10 cameras mounted perpendicularly on an aluminum frame, looking at the tank from top and side view (Fig 1). The cameras were connected to the host computer via USB ports. Video capture and camera control were conducted using an in-house Python script “goproUSB”, which we made available as an open-source software [18]. The software allowed precise time synchronization of the video capture processes. At the edges of the tank we attached eight magnetic markers–four at the water surface, and four other 100 mm below. The markers determined two parallel reference planes for each camera and were used for calibration. We extracted fish contours from video recordings based on HSV color filtering. Locations of the centers of gravity in each image were determined in coordinates corresponding to all the reference planes. Based on the data from both cameras we performed triangulation, obtaining fish position in 3D space. The detailed description of the localization algorithm is presented in the S1 Appendix. Using time stamps assigned to all the data points we merged the fish coordinates with the corresponding, measured impedance values. The operation of the system is illustrated in Fig 2.

### Fish detection algorithm

The main aim of the current study was to detect fish passes between the electrodes based on the impedance measurements only. Due to the higher electrical conductivity of fish tissues than the surrounding water, and due to their capacitive character, fish appearance in the detection region should manifest itself by observable decays in both real and imaginary impedance components. The extent of this decay may vary significantly due to e.g. fish distance from the electrodes, body orientation, and other factors. Reliable detection of fish-induced signals in the presence of background noise, for example related to fluctuations of electrical properties of water is a major challenge in the fish counting process.

Previous studies evaluating resistivity counters utilized resistance changes only, and moreover, as the detection was performed by commercial devices, it remained unclear how the noise and variable water resistivity issues were handled. As a result, these studies provided little information on potential sources of error, and for optimization.

**Fig 3:**
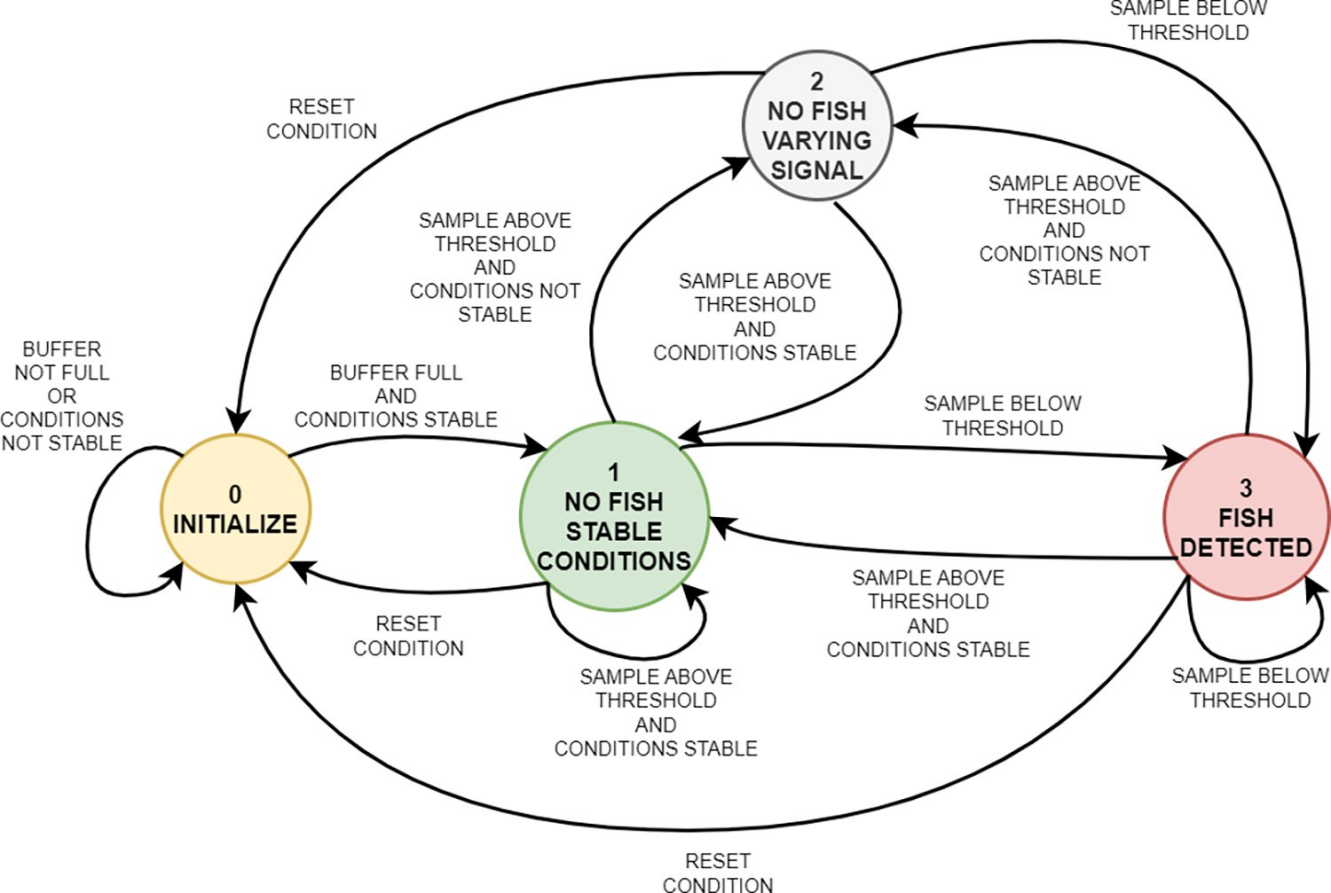
Finite state machine diagram of the fish detection algorithm. The state of the system switches between four possible states (numbered from 0 to 3), based on the current state of the system and on new samples coming in. Descriptions next to arrows present the corresponding transition conditions.

Here, we describe and evaluate a detection algorithm, based on a finite state machine (FSM) model, intended for counting fish in real time based on impedance measurements only (Fig 3).

Every step of the detection process starts with reading an impedance data sample into a buffer of 200 samples. Variations in the buffered signal and the value of a new sample determine how the system switches between states. It remains in the initial state (0) until the data buffer is full and for as long as conditions are unstable. The stability criterion included a maximum allowed signal swing (100 mΩ for resistance and 100 μΩ for reactance) and a maximum slope (2.5 mΩ/s for resistance and 10 μΩ/s for reactance), based on the buffered data. Once the buffer is full and the criterion is fulfilled, the system switches to state (1), meaning that no fish is detected, and the operating conditions are stable. The system switches to the fish detected state (3) when a new sample is below a specified threshold value related to a temporal noise level. To define this noise level, we take advantage of the fact that in freshwater fish presence should manifest itself by signal decays. Thus, all the positive signal peaks are considered to be manifestations of noise. We calculate the current noise amplitude as the maximum positive deviation from the median value of the buffered data. A fish is detected if a value of an acquired sample is lower than the signal median minus a set threshold ratio times the peak-to-peak noise level (i.e., doubled noise level amplitude). A threshold ratio of 0,5 means that any signal drop below the temporal noise level would be interpreted as fish appearance. The higher the threshold ratio, the larger the signal drop required for the system to switch to the fish detected state. If a fish is not detected, but signal statistics temporarily exceed the stability criteria, the system switches to state (2), in which there is signal variation surpassing the noise, but no fish present. In this state, the noise threshold level is not updated but remains equal to that for the last stable period. This approach performs automatic adjustment to slow variations and avoids errors due to incidental, excessive noise events–e.g., due to electromagnetic interference. In case of unpredicted system failures–such as longer pauses between subsequent data samples, the system performs an internal reset and switches back to the initial state (0).

The detection algorithm can operate in four different modes:

- Mode “re”–only the resistance values are considered
- Mode “im”–only the reactance values are considered
- Mode “or”–the transition between states is performed based on logical disjunction of condition outcomes independently calculated for resistance and reactance values
- Mode “and”–the transition between states is performed based on logical conjunction of condition outcomes independently calculated for resistance and reactance values

We extensively tested all operating modes, with different threshold ratio values. Hereto we used the recorded measurements, feeding the data to the algorithm input one sample at a time. This approach mimics real-time operation, while enabling the use of exactly the same datasets for evaluating fish counting performance versus different system settings.

### Numerical model and simulations

To thoroughly understand impedance-based fish detection and guide the design of electrode configurations, we developed a finite-element numerical model of the experimental setup, including a simplified fish model. The simulations were conducted using Comsol Multiphysics software controlled by in-house Python scripts to perform complex parameter sweepings for experimentally recorded fish tracks. We approximated the measurement electrodes with a two-electrode setup consisting of rectangular stripes. Such an approximation is justified because numerical models do not take ionic double-layer formation into account, and a pair of comb-like PU and CC electrodes can, in this regard, be treated as a single electrode. The size and shape of the model domain resembled the water volume inside the tank. The fish was modelled as an ellipsoid with axes equal 50 mm, 20 mm, and 10 mm. It was assumed that the electrical conductivity of fish is homogeneous and equal to 1 S/m. Electrical conductivity of water was adjusted for each measurement using a single data point for which fish was away from the electrodes, so the calculated resistance in such a case would be within ±50 mΩ accuracy with the experimental data.

First, the model was qualitatively validated using the experimental data. We extracted single fish tracks corresponding to a fish passage from one side of the tank to the opposite side, passing in between the electrodes. The track consisted of subsequent fish coordinates with assigned measured impedance values and time stamps. We smoothed the track using the LOWESS algorithm [19, 20] independently on all three spatial coordinates versus time. Next, we determined the spatial orientation of the fish for every point in space, based on the vector pointing to the subsequent point in the track. Without smoothing this operation would result in excessive errors in direction estimation. Model simulations were run for each location of the fish, taking body orientation into account, where orientation was assumed to be parallel to the direction of motion. We compared the overall range and dynamics of predicted and measured resistance changes to test if the results of simulations resemble the results of experiments.

After validation of the model based on experimentally recorded tracks, the model was used to systematically map the resistance value distribution versus fish location in a horizontal cross-section of the tank.

Apart from simulations including the simplified fish model we also calculated spatial distribution of impedance sensitivity as a function of geometrical configuration of the electrodes. The impedance sensitivity describes a contribution of each voxel of the model to the total calculated impedance, and is defined with the following relation [13, 14, 21]:

$$S=\frac{\vec{J_{PU}}\cdot \vec{J_{CC}}}{I^{2}},$$ (3)

where $\vec{J_{PU}}$ and $\vec{J_{CC}}$ are the current density vectors corresponding to current flows between PU and CC electrodes, respectively, and *I* is the set current intensity.

For the latter simulations the impedance sensitivity is determined without taking the presence of a fish into account, and its spatial distribution can be considered as an estimate of relative changes in the signal caused by the fish passing through a specific volumetric region.

## Results

### Detection performance

**Fig 4:**
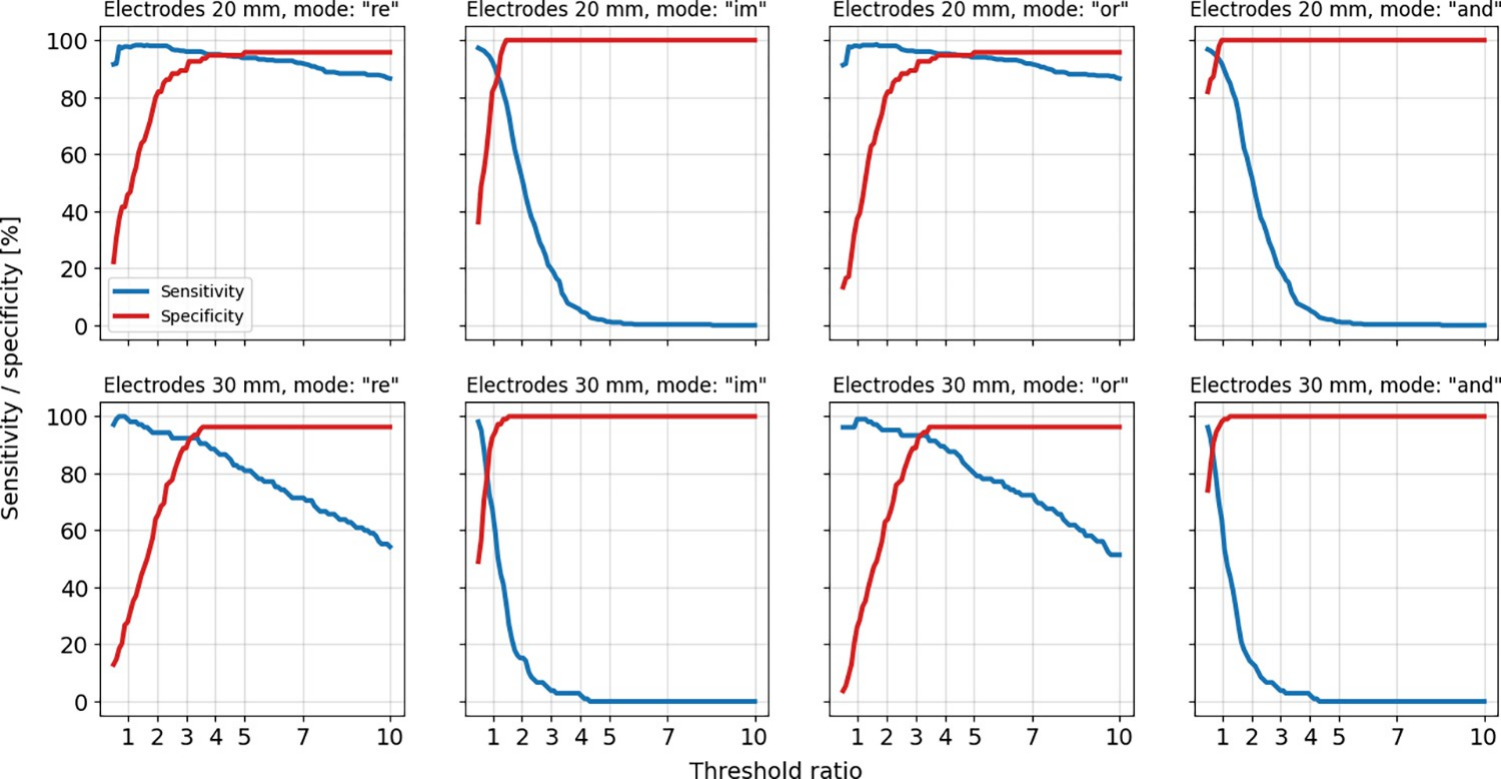
Detection sensitivity and specificity. Detection system sensitivity plots (in blue) and specificity plots (in red) determined for various system settings and two electrode configurations: 20 mm (top row) and 30 mm (bottom row). Tracks representing fish passes between the electrodes and fish absence were extracted from approximately 3-hour long recordings for each of the electrode configurations.

To quantitatively evaluate detection performance for different settings we collected approximately 3 hours of impedance recordings and corresponding fish localization data for both the 20 mm and 30 mm wide electrode configurations. From these data sets we extracted tracks corresponding to actual passes (from within 50 mm to one side wall of the tank to within 50 mm of the opposite side wall) and tracks where the fish remained near one of the side walls (within 50 mm) for at least 10 s. For the former tracks the detection system should yield a hit, whereas the latter should yield no fish detection. Impedance values for each track were fed into the FSM model, and outcomes simulating actual passes were classified as ‘true positives’ if the FSM state changed from no fish to fish detected, and as ‘false negatives’ otherwise. Similarly, fish tracks without electrode passes were recognized as “true negative” if the state history did not include any “fish detected” states, or as “false positive” otherwise. We then calculated the system sensitivity and specificity using the following formulas:

$$sensitivity=\frac{TP}{TP+FN},$$ (4)

$$specificity=\frac{TN}{TN+FP},$$ (5)

where TP, FN, TN, and FP denote the numbers of true positive, false negative, true negative, and false positive events, respectively. The results obtained for different operation modes of the FSM model and for different threshold ratio settings are presented in Fig 4. Specificities increase to nearly 100% for increasing threshold ratios. Sensitivities decline for increasing threshold values. An optimal configuration should demonstrate both high sensitivity and specificity values. Such outcomes are achieved for detector modes „re”and „or“. Results for the 20- and 30-mm wide electrode configurations are comparable, the main difference being a more rapid decay of sensitivity with increasing threshold ratio for 30 mm electrodes.

**Fig 5:**
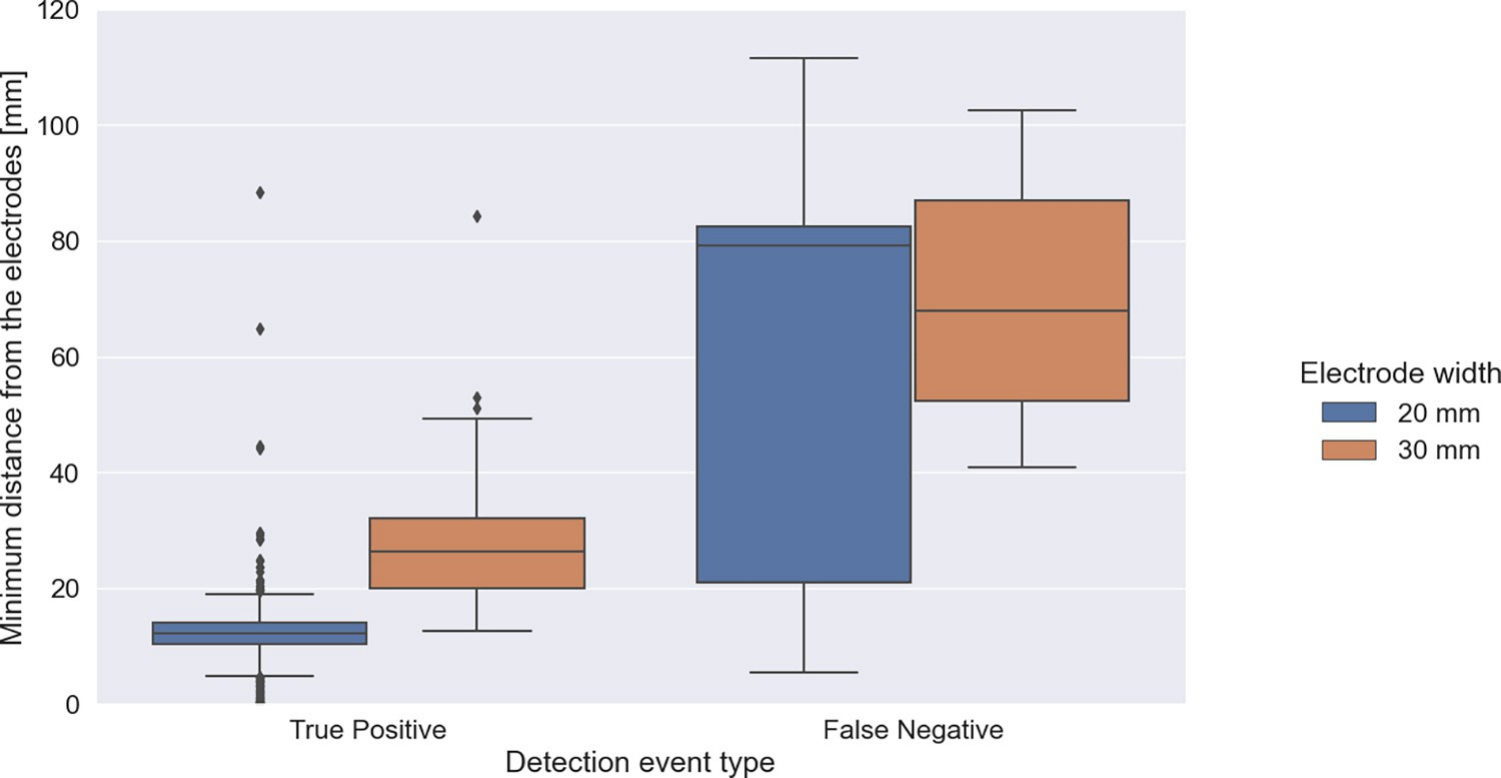
Fish detection events classification. Box plots presenting minimum distances of the fish from the measurement electrodes while passing between them for detection events classified as true positives and false negatives. Blue boxes present results obtained for 20 mm wide electrodes, while orange boxes–for 30 mm wide electrodes.

For threshold ratios in the range between approximately 3 and 4 the determined sensitivity and specificity values exceed 90%, for „re”and „or”operating modes. False negatives were due to various factors, for example the minimum distance of a track relative to one of the electrodes (see Fig 5). Compared to true positives the false negative tracks pass at substantially larger distance from the electrode.

**Fig 6:**
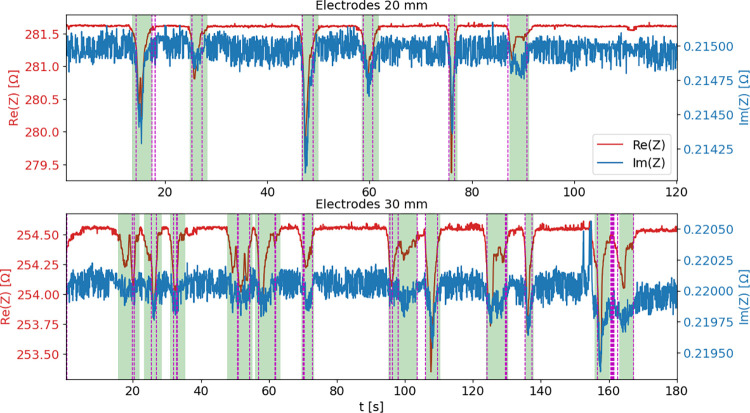
Fish detection events. Resistance plots (in red) and reactance plots (in blue). The detection events determined based on impedance measurements are marked with green background, while fish passes determined based on fish localization reconstructed from the synchronized video recordings are marked with vertical dashed lines.

Fig 6 presents an example of results of fish detection in the tank for two short measurements conducted using electrodes 20 mm and 30 mm wide. Plots illustrate resistance and reactance waveforms versus time, with marked detection events based on impedance measurements (green background) and reference video recordings (vertical dashed lines). The FSM fish detector in this example was operating in mode “re” (utilizing resistance data only), with the threshold ratio set to 2. For the reference video analysis it was assumed that the fish was detected within a distance of 50 mm relative to the centers of the electrodes. The output of the finite state machine, based on the impedance detector, is in near perfect agreement with the visual observations, with clear, observable decays in the measured values corresponding to fish passes between the electrodes.

#### Numerical simulations

**Fig 7:**
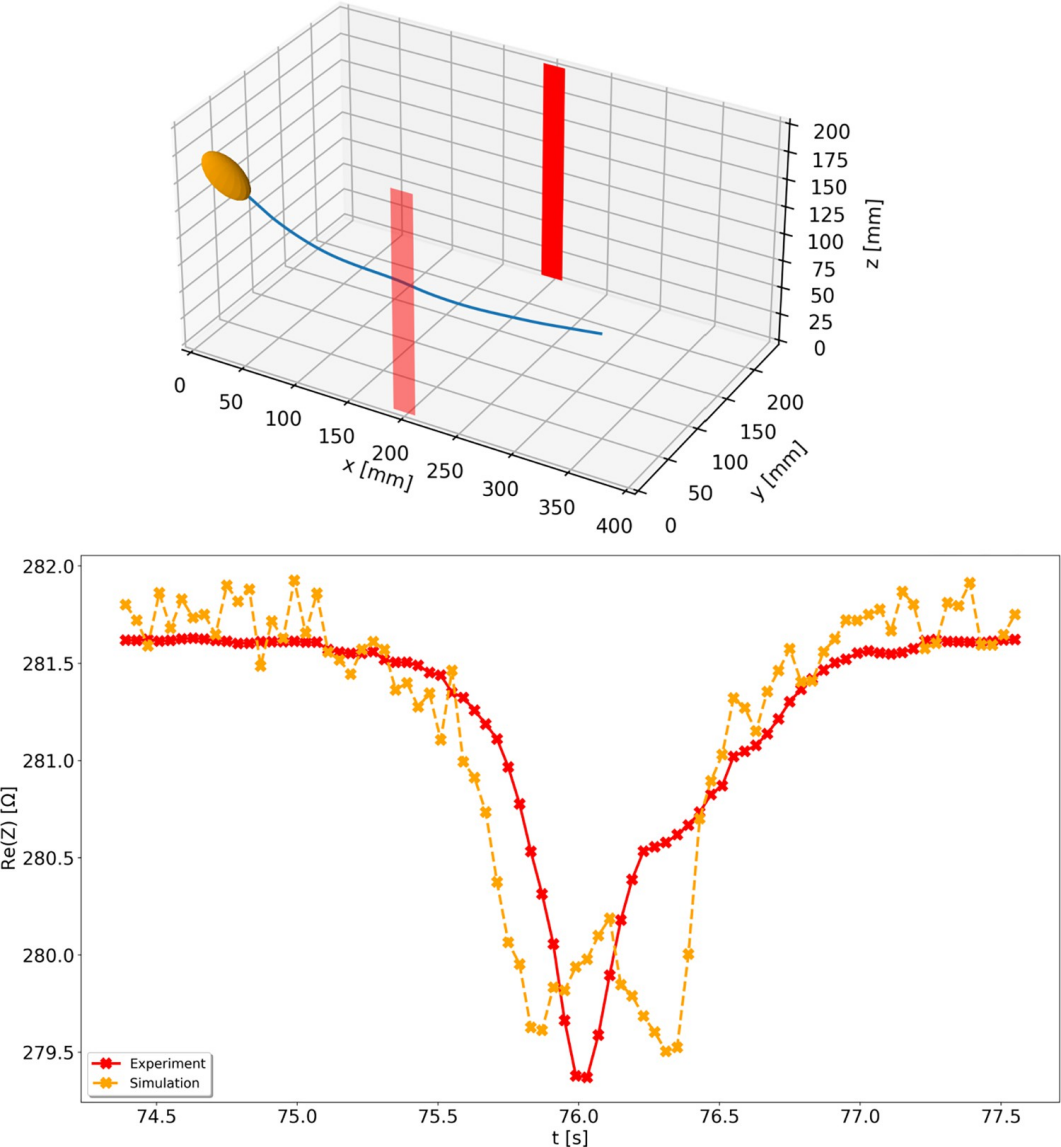
Changes in resistance values determined during a fish pass. A fish track extracted from experimental measurements with simplified ellipsoidal fish model and electrodes depicted in red (top plot), and resistance values determined experimentally and numerically as functions of time during a fish pass along the indicated track (bottom plot).

To explain and better understand the observed phenomena we employed a finite element numerical model. We first qualitatively validated the model by performing simulations using fish tracks extracted from experimental measurements and then comparing the resistance values obtained numerically and experimentally. An example of the results is presented in Fig 7. No attempt was made to fit the model to the data, except for adjusting the water resistivity to match experimental and simulated data at the beginning of a track, when the fish was away from the electrodes and did not affect the readings. Although we used a highly simplified fish model (see methods), the simulated resistance curve fairly resembles the measured changes, especially in terms of the expected signal drop range. The example in Fig 7 also shows a clear discrepancy; the simulation shows a double minimum whereas the experimental curve has a single local minimum. Such discrepancies are to be expected due to the simplified fish that did not take body shape into account. In close proximity of an electrode small deviations in fish orientation and body shape (tail beat) might result in large, observable changes.

**Fig 8:**
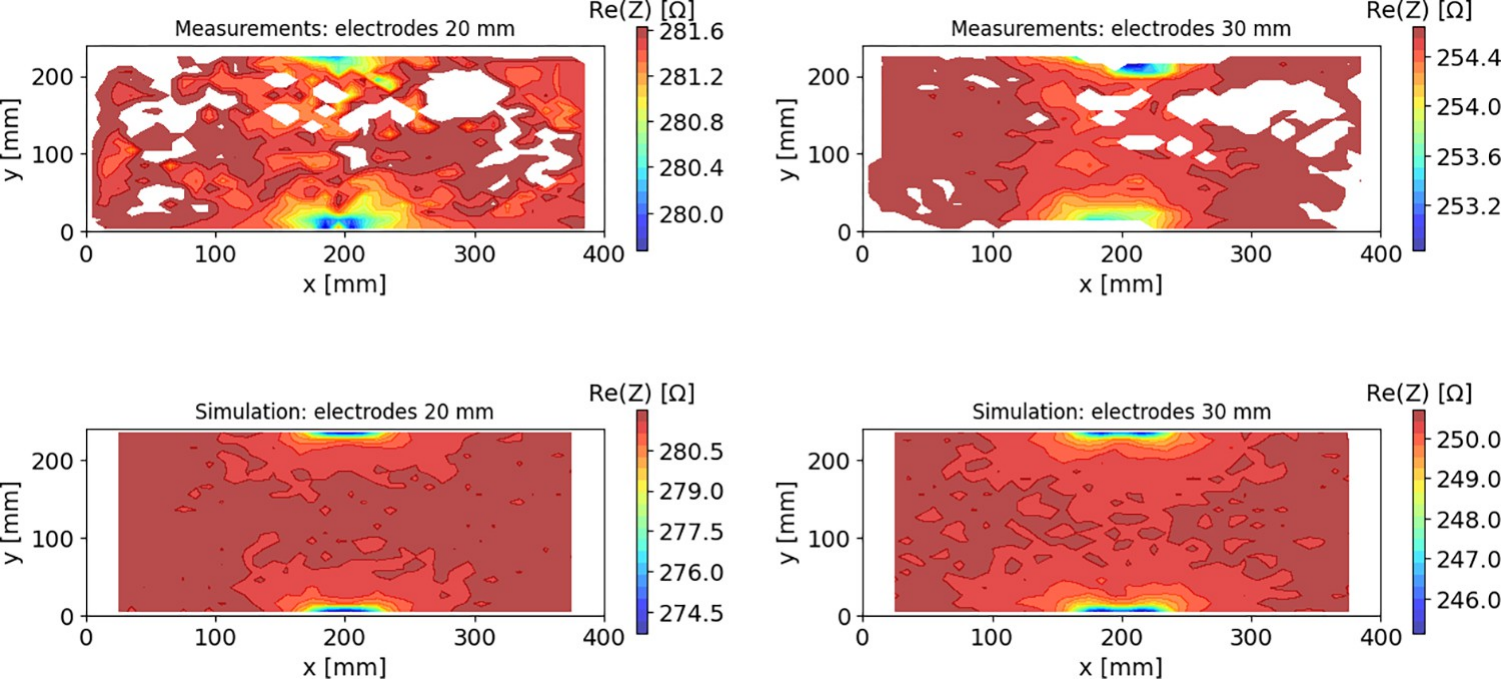
Resistance values as functions of fish locations in the cross-section of the tank. Resistance values as functions of fish locations in the cross-section of the tank: measurements (top row) and simulations (bottom row) determined for electrode widths 20 mm (left column) and 30 mm (right column). Simulation results were obtained for a 10 mm grid resolution, at a fixed depth of 100 mm.

**Impedance sensitivity spatial distribution.** After establishing that the finite element model correctly predicts the amplitude of impedance modulations, we used the model to systematically determine sensitivity as a function of fish location in the tank. We swept the model fish location across a grid in the xy plane of the tank, at a depth of 100 mm, assuming constant orientation of fish body (horizontal and parallel to the longest tank edge). To compare these modelling results to experimental data we aggregated the experimental results based on fish xy coordinates and calculated median values for spatial bins of 10 x 10 mm. The results for model simulations and for experimental data are presented in Fig 8. White spots correspond to regions where the fish never passed during the recordings. Both numerical predictions and experimental results demonstrate highly non-uniform spatial sensitivity of the detection system to the presence of the fish (expressed by the observed impedance changes), with significant hot-spots and steep gradients in the vicinity of the electrodes. Near such hot spots, small deviations in geometry settings might lead to relatively large changes. The overall measured and calculated shapes of resistance distributions are, however, in fair agreement, and the model clearly reproduces the differences observed experimentally between the 20- and 30-mm electrode configurations, and the decline of sensitivity at larger distances from the electrodes. This also explains the system behavior illustrated in Fig 5.

**Fig 9:**
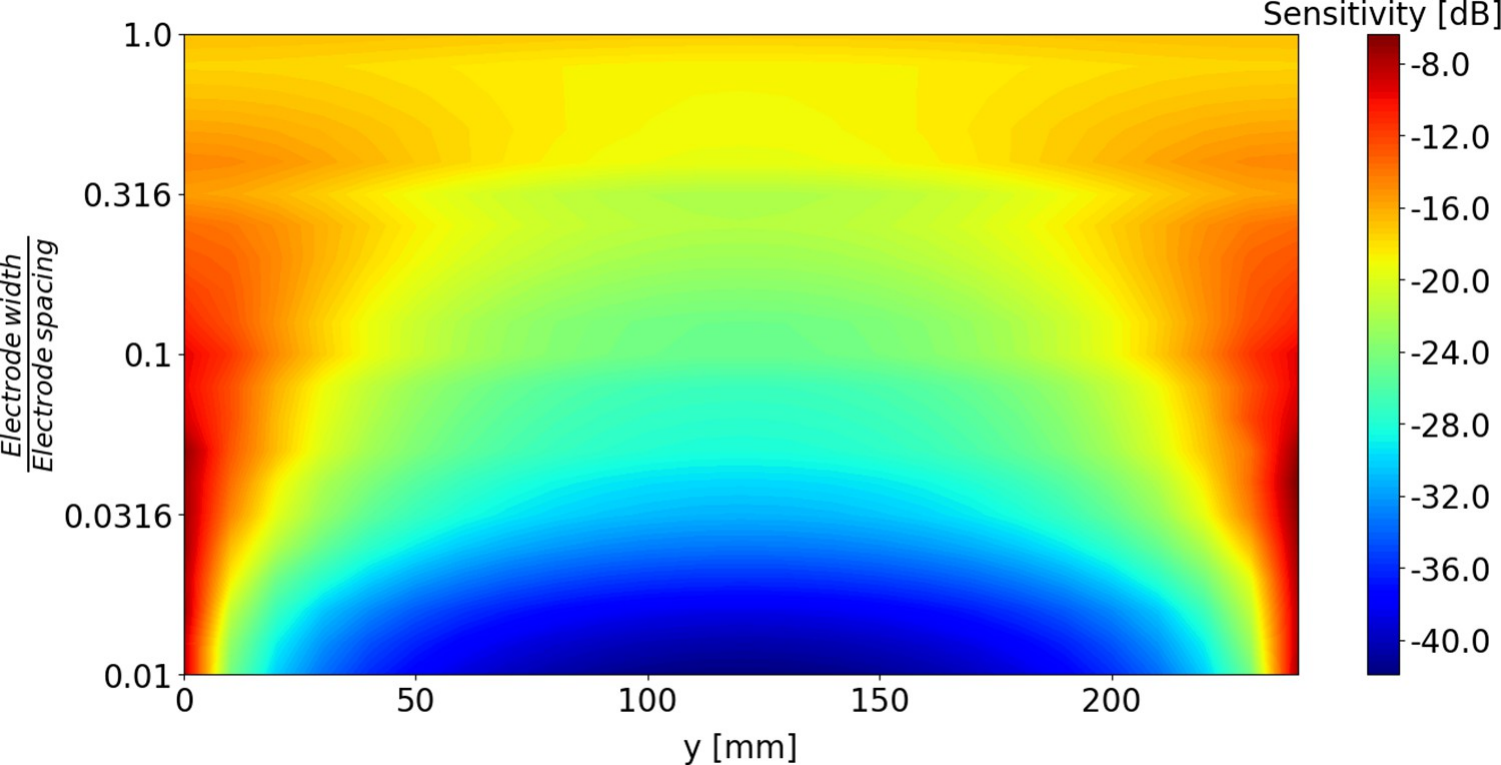
Impedance sensitivity distributions. Impedance sensitivity distributions (expressed in dB scale) along the line between middle of the electrodes plotted versus electrode width to spacing ratios.

The results presented in Fig 8 demonstrate sensitivity profiles vary with both the width of the electrodes, and with the distance relative to the electrodes. Simulations with a model fish at different locations nicely reproduce these findings. The model can, therefore, be used to characterize sensitivity profiles in a general way, i.e., independent of the actual experimental settings. Hereto, we calculated the impedance sensitivity distribution with Eq (3) (i.e., the contribution of each volume element to the overall determined impedance value) for different ratios of electrode widths to electrode spacing. The resulting sensitivity profiles are presented in Fig 9. For narrow electrodes, compared to the distance between them, we see distinct hot spots in the direct vicinity of the electrodes and lower impedance sensitivity in the middle part. Very narrow electrodes at large distance therefore may have a blind spot in the middle, where fish passes are unlikely to be counted. Increasing the electrode widths while preserving the spacing leads to flattening out the impedance sensitivity distribution, with less distinct hot spots at the edges and with higher sensitivity in the middle part. In such a case we would expect higher observable changes in measured resistance for fish passes through the central part, but lower signal amplitudes for passes close to the electrodes, compared to an electrode configuration with lower width to spacing ratio. In the extreme case of very wide electrodes the impedance sensitivity would be uniformly distributed between them, resulting in a signal that would be almost independent of the fish trajectory, but the changes in signal introduced by the fish presence would be rather low.

## Discussion

To study the fundamentals of fish detection by means of electrical impedance measurements we conducted laboratory experiments using a fish passage setup in artificial water tank and combined it with detailed numerical simulations. Our main aim was to provide a theoretical background, and a tool for designing impedance-based fish counting systems for real-world applications. The glass tank with dielectric walls constitutes a fair model of small fish pass insets used in combination with various commercial counters. Further upscaled, it might correspond to larger artificial fish passages. Some of the findings reported herein have a more general character, independent on the exact geometry of the system. This includes the description of the detection algorithm, the hardware configuration utilizing a low-cost AFE, using numerical models for system design and optimization, and the issues related to non-uniform impedance sensitivity distribution between the electrodes. Such topics were not covered by the studies presented in the literature, which focused on assessing detection performance of specific setups utilizing commercial fish counters.

Our detection algorithm, based on a finite state machine operating on incoming measurement samples, achieved sensitivity and specificity of over 90%. We also demonstrated that most of the misidentified events concerned fish passes through the central part of the tank, where detection performance was worst due to the non-uniform current density distribution between the electrodes. The effects of such non-uniform distributions were further analyzed using model simulations, that allowed us to characterize the trade-off between maximal sensitivity close to the electrodes and sufficient sensitivity in the middle of the electrode pairs. These observations suggest potential methods for improvement, or for adjusting the configuration to specified demands. In this regard it would be beneficial to keep the dimensions of a fish pass used for counting as small as possible, while still enabling all the fish to swim through. The fish used for experimental investigations described here was relatively small compared to the sizes of the tank and the electrodes. For larger fish–as it was also demonstrated in other studies in the literature [9, 11]–the detection performance should be substantially higher.

All described experiments were conducted using a low-cost AFE and a microcontroller, utilizing in-house software. Our results demonstrate that such means can constitute an efficient alternative to expensive commercial devices for detecting fish in water. As presented in Figs 4 and 7, the resistance waveforms show high signal to noise ratio and electrical characteristics of the hardware used do not seem to limit the detection performance. Using in-house software enables complete control over the data acquisition and processing, providing also multiple possibilities for further improvements. The parameters of such a system can be easily adjusted to specific requirements imposed by environmental conditions. Due to the low cost and using standard serial port interface for the data transfer it is also possible to use multiple measurement devices together, forming a multi-channel fish counting system. Such an approach could be used to extend the detection region or to provide additional information on fish size, velocity, or direction of movement.

The finite-element numerical model including a simplified fish model proved to be an efficient tool in predicting measured impedance changes due to the fish presence between the electrodes. The results of numerical simulations enabled us to explain and understand the observed phenomena, such as the relation between electrode shape and spacing and the detection sensitivity distribution. Numerical calculations are much faster and more cost-efficient than iterative experimental measurements, and thus can be used for adjustments and optimization of a fish counting system for specific environmental conditions and detection demands.

The highest detection performance in our study was achieved when using either resistance data only, or when combining resistance and reactance data by logical disjunction. Including the imaginary part of impedance did, however, not result in a significant increase in sensitivity or specificity. Still, it might be useful in distinguishing between fish and, e.g., artificial or non-living objects. In our previous study we demonstrated that reactance, in contrast to resistance, was not significantly affected by the presence of air bubbles in the detection region [13]. As reactance measurements come at no extra cost, the combination of resistance and reactance could aid in off-line identification of objects, after the detection algorithm.

The introduced fish counting system could be used independently, but it can also benefit from combination with a visual observation setup. In the latter case a detection event would trigger a camera to take a static picture of a detected object passing between the electrodes. Such a combination would allow to further improve detection performance and provide additional information regarding fish species and size. Compared to a video-only based fish counting system, there would be no need for real-time fish detection in video recordings, which would significantly decrease the required computational power, system complexity and cost.

In the described experiments a single fish was used to study fundamentals of electrical impedance based detection. Under real-world conditions it is likely that multiple fish might appear simultaneously in the detection region. The outcomes of our studies provide cues on how such an issue can be addressed. One of the possible solutions is to use supplementary cameras triggered by the identified detection events, as described in the previous paragraph. In stand-alone operation, without additional cameras, the problem of multiple fish occurrences might be resolved, e.g., by narrowing passages between the electrodes and/or multiplying the measurement channels (i.e., utilizing independent electrode pairs), so that only one fish could pass between a single electrode pair at a time. It is also possible that further studies on signal processing means will eventually allow to extract and separate the information on detected individuals based on single channel impedance recordings only.

## Conclusion

We introduced a complete framework for design and construction of an impedance-based fish counting system using parallel strip electrodes arranged in cross-section of a water stream. The framework includes a hardware setup utilizing a low-cost analog front-end and a general-purpose microcontroller, a detection algorithm with adaptive thresholding, described in the form of a finite state machine, and a finite-element numerical model enabling accurate predictions of measured resistance values. We investigated the system characteristics and phenomena underlying fish detection using the described approach. We demonstrated that detection sensitivity and specificity determined under laboratory conditions exceed 90% for a fish significantly smaller than distance between the electrodes. We found the main factor limiting the achievable detection performance to be the non-uniform impedance sensitivity distribution between the electrodes. Most of the false negative detection events occurred when fish was passing close to the middle of the tank and away from the electrodes. This indicates the need of adjusting the system geometry to requirements and conditions imposed by specific applications–e.g., by narrowing the space between the electrodes and adjusting electrode width to spacing ratio. This optimization process can be efficiently aided with simulations using the introduced model.

## Supporting information

S1 Video An example video presenting measurement setup operation. Recordings from both cameras with determined and marked fish contours (top) and synchronized impedance plots showing resistance values versus fish position in 3D space (bottom left) and resistance and reactance values versus time (bottom right).
(MP4)

S1 Appendix Appendix: Retrieving fish coordinates in 3D space from video recordings. Detailed description of the fish localization algorithm based on images extracted from camera recordings.
(PDF)
