# Supplementary material for: Understanding and optimizing fish counting techniques based on electrical impedance measurements
Source: PLoS One. 2023 Oct 30;18(10):e0293699. doi: 10.1371/journal.pone.0293699 (PMC10615265; doi:10.1371/journal.pone.0293699)
Supplement: S1 Appendix — Detailed description of the fish localization algorithm based on images extracted from camera recordings. (PDF) [file pone.0293699.s002.pdf]

Appendix: retrieving fish coordinates in 3D space from video recordings.

Lukasz J. Nowak, Martin Lankheet

Notation

p_r^x - real-world x-coordinate, expressed in $[m]$
p_r^y - real-world y-coordinate, expressed in $[m]$
p_r^z - real-world z-coordinate, expressed in $[m]$
p_p^x - pixel-wise x-coordinate. Determined from the image, expressed in pixels
p_p^y - pixel-wise y-coordinate. Determined from the image, expressed in pixels
p_p^z - pixel-wise z-coordinate. Determined from the image, expressed in pixels

2,5-D transformation algorithm

The fish localization algorithm utilizes two cameras looking at the tank from two perpendicular angles: from top and side of the tank. The cameras are synchronized, so that corresponding frames in the corresponding recordings are assumed to present snapshots of the corresponding moments in time. Here, it is assumed that Camera 1 is recording from the top (its sensor is arranged in XY plane), and Camera 2 is capturing the side view of the tank (its sensor is arranged in YZ plane). We used two GoPro Hero10 cameras operating in Linear mode. It was assumed that non-linear distortions remaining after the calibration procedure are relatively small compared to fish dimensions and accuracy of coordinates detection based on contour identification.

In order to determine fish location inside the tank reference points with known real-world coordinates are needed (e.g., relative to one of the tank corners). Here, we use reference points arranged in two parallel planes (parallel to themselves and to camera sensor planes). There are four points per plane, forming vertices of rectangles on the tank walls. In the experimental setup we used a small magnetic balls painted in bright yellow, which were later on marked on camera frames to read their pixel coordinates on images from both cameras. We also knew their real-world coordinates inside the tank. This is illustrated in Figure 1. Points corresponding to two reference planes for each camera are marked in blue and red.

The localization algorithm consists of the following steps:

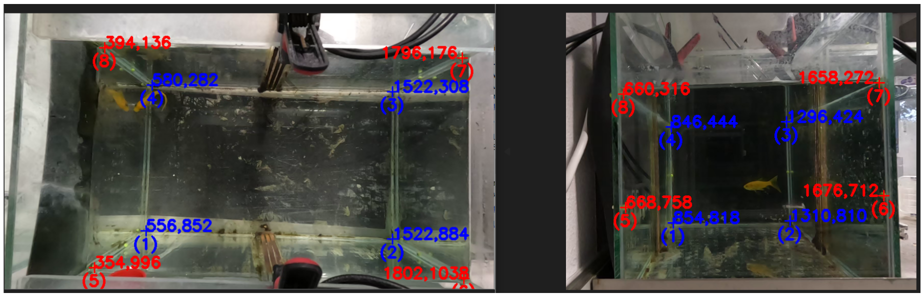

Figure 1: Reference points inside the tank with their pixel coordinates on the images. Numbers in brackets are the points' indices. The points form two sets of vertices of rectangles, defining two reference planes (marked in blue and red) for each camera view. Point 1 is the origin with real-world coordinates (p_r^x, p_r^y, p_r^z) = (0,0,0).

1. Corresponding images from both cameras are loaded, analyzed and pixel coordinates of the point of interest (i.e., fish location) are extracted
2. Pixel coordinates of a point of interest in both images are converted into real-world coordinates in both reference planes for each camera (thus, 2D coordinates + known third coordinate of a specific plane; such a procedure, with fixed and discrete set of one of the coordinates is referred to as 2,5D transformation)
3. Cooefficients in 3D real-world coordinates of lines connecting points in refernce planes are determined for each camera
4. 3-D real-world coordinates of the point of interest are determined as the intersection ponint (closest-distance point) between the lines

Determining fish localization in the video frames.

Video frames are processed one by one, utilizing the procedure described herein to each single image pair. First, the frames are color-filtered by applying HSV (hue, saturation, value) thresholds to each image. The threshold values were determined manually on randomly selected frames, so that only the fish was visible in the filtered images. They are specific for given optical properties of fish, background, and lightning conditions. Any residual artifacts were significantly smaller than the fish size. The procedure is illustrated in Figure 2.

The color filtering procedure does not remove fish reflections from the glass tank walls. However, they can be easily identified and removed afterwards, by selecting only the object closest to the middle of the tank.

Fish contours are extracted from the processed images using OpenCV library in Python and *cv2.findContours()* function. Fish coordinates (pixel-wise) are determined as coordinates of the center of gravity of the contour.

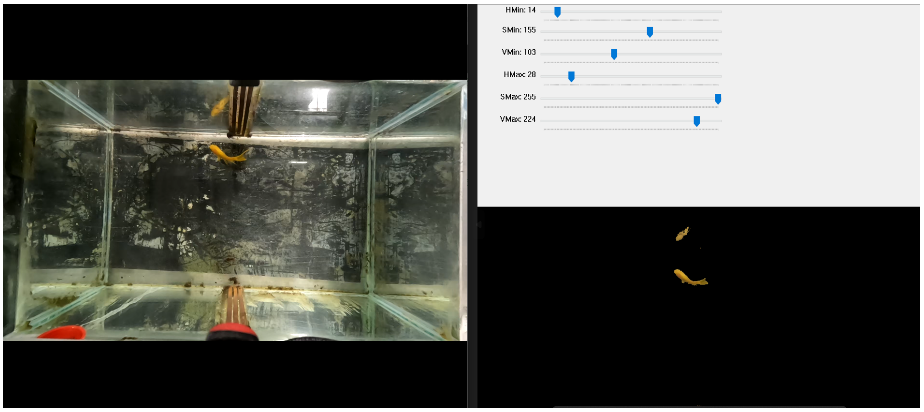

Figure 2: A captured video frame from Camera 1 (left), and the same frame after applying HSV color filtering (right). Only the fish and its reflection from the wall are visible.

Transformation between pixel and real-world coordinates

It is assumed that the cameras are perfectly perpendicular and look straight into the parallel reference planes. It is also assumed that the transformation between pixel and real-world coordinate system includes translation, rotation and scaling. The transformation matrices and equation can be expressed as follows:

$$\overrightarrow{p_r} = \overline{TRS}\,\overrightarrow{p_p}, \tag{1}$$

where:

$$\overrightarrow{p_r} = \begin{bmatrix} p_r^x \\ p_r^y \\ 1 \end{bmatrix} \tag{2}$$

is the real-world coordinate vector for Camera 1 and a given reference plane. The corresponding vector for Camera 2 will only differ in superscripts, having yz instead of xy coordinates. The p_r^z coordinates of the reference points for Camera 1 and p_r^x coordinates of the reference points for Camera 2 are known (from the set locations of the markers).

$$\overrightarrow{p_p} = \begin{bmatrix} p_p^x \\ p_p^y \\ 1 \end{bmatrix} \tag{3}$$

is the pixel coordinate vector for camera no. 1, and:

$$\overline{TRS} = \overline{T}\,\overline{R}\,\overline{S} \tag{4}$$

is the transformation matrix, being multiplication product of translation $\overline{T}$, rotation $\overline{R}$, and scaling $\overline{S}$ matrices, expressed with the following equations:

$$\overline{T} = \begin{bmatrix} 1 & 0 & t_x \\ 0 & 1 & t_y \\ 0 & 0 & 1 \end{bmatrix}, \tag{5}$$

$$\overline{R} = \begin{bmatrix} cos\theta & -sin\theta & 0 \\ sin\theta & cos\theta & 0 \\ 0 & 0 & 1 \end{bmatrix}, \tag{6}$$

$$\overline{S} = \begin{bmatrix} s & 0 & 0 \\ 0 & s & 0 \\ 0 & 0 & 1 \end{bmatrix}, \tag{7}$$

where t_x and t_y are translation coefficients, θ is rotation angle between the coordinate systems, and s is the scaling factor.

Thus:

$$\overline{TRS} = \begin{bmatrix} A & -B & t_x \\ B & A & t_y \\ 0 & 0 & 1 \end{bmatrix}, \tag{8}$$

where $A = s\,cos\theta$, and $B = s\,sin\theta$.

Thus, we can rewrite the equation (3) as:

$$\begin{bmatrix} p_r^x \\ p_r^y \\ 1 \end{bmatrix} = \begin{bmatrix} A & -B & t_x \\ B & A & t_y \\ 0 & 0 & 1 \end{bmatrix} \begin{bmatrix} p_p^x \\ p_p^y \\ 1 \end{bmatrix} = \begin{bmatrix} A\,p_p^x - B\,p_p^y + t_x \\ B\,p_p^x + A\,p_p^y + t_y \\ 1 \end{bmatrix} \tag{9}$$

In order to determine real-world fish coordinates we first calculate A, B, t_x, and t_y, based on the known real-world and pixel coordinates of the reference points in the tank (Figure 1). The calculations of the sought $\overline{TRS}$ matrices' coefficients need to be performed independently for Camera 1 and Camera 2, and for the corresponding reference planes. Generalizing the problem into n reference points for each reference plane and assuming $n \geq 2$, we use linear least squares (LLS) method and the following matrix equations (xy coordinates refer to Camera 1. For Camera 2 they are replaced with the corresponding yz coordinates):

$$\overline{X_P}\,\overrightarrow{M} = \overrightarrow{X_R} \tag{10}$$

where:

$$\overline{X_P} = \begin{bmatrix} p_{p1}^x & -p_{p1}^y & 1 & 0 \\ p_{p1}^y & p_{p1}^x & 0 & 1 \\ p_{p2}^x & -p_{p2}^y & 1 & 0 \\ p_{p2}^y & p_{p2}^x & 0 & 1 \\ ... & ... & ... & ... \\ ... & ... & ... & ... \\ p_{pn}^x & -p_{pn}^y & 1 & 0 \\ p_{pn}^y & p_{pn}^x & 0 & 1 \end{bmatrix} \tag{11}$$

is the pixel-coordinate matrix of reference points, with dimensions $2n \times 4$ (n is the number of reference points in a given plane). p_{pm}^{x} is the x pixel-coordinate of the reference point no. m.

$\overrightarrow{X_R}$ is the vector of the corresponding real-world coordinates of the n reference points:

$$\overrightarrow{X_R} = \begin{bmatrix} p_{r1}^{x} \\ p_{r1}^{y} \\ p_{r2}^{x} \\ p_{r2}^{y} \\ \cdots \\ \cdots \\ p_{rn}^{x} \\ p_{rn}^{y} \end{bmatrix}, \tag{12}$$

and $\overrightarrow{M}$ is the vector of the sought transformation coefficients:

$$\overrightarrow{M} = \begin{bmatrix} A \\ B \\ t_x \\ t_y \end{bmatrix}, \tag{13}$$

Under such conditions we can approximate $\overrightarrow{M}$ with its best match $\overrightarrow{M^*}$ based on the LLS principle:

$$\overrightarrow{M^*} = \underline{X_P}^T \left(\underline{X_P}\,\underline{X_P}^T\right)^{-1} \overrightarrow{X_R} \tag{14}$$

Point localization in 3D space

The pixel coordinates of the fish read from the corresponding frames from Camera 1 and Camera 2 are first converted into four points corresponding to the coordinates in all four reference planes (i.e., two for each camera view). This is done using equation (1) with matrix coefficients calculated using equation (14) and the reference points' coordinates. The result are four points defined with real-world coordinates corresponding to four reference planes:

$$\begin{gathered} \overrightarrow{r_1^{cam1}} \left(r_{1x}^{cam1}, r_{1y}^{cam1}, r_{1z}^{cam1}\right), \\ \overrightarrow{r_2^{cam1}} \left(r_{2x}^{cam1}, r_{2y}^{cam1}, r_{2z}^{cam1}\right), \\ \overrightarrow{r_1^{cam2}} \left(r_{1x}^{cam2}, r_{1y}^{cam2}, r_{1z}^{cam2}\right), \\ \overrightarrow{r_2^{cam2}} \left(r_{2x}^{cam2}, r_{2y}^{cam2}, r_{2z}^{cam2}\right), \end{gathered} \tag{15}$$

where subscripts denote point numbers (corresponding to reference plane numbers) and superscripts denote camera indices. A pair of points for each camera defines a line, which can be defined with the following vector equations:

$$\begin{aligned} L_1 : \overrightarrow{v_1} &= (r_{1x}^{cam1}, r_{1y}^{cam1}, r_{1z}^{cam1}) + l_1\,(r_{2x}^{cam1} - r_{1x}^{cam1}, r_{2y}^{cam1} - r_{1y}^{cam1}, r_{2z}^{cam1} - r_{1z}^{cam1}) \\ L_2 : \overrightarrow{v_2} &= (r_{1x}^{cam2}, r_{1y}^{cam2}, r_{1z}^{cam2}) + l_2\,(r_{2x}^{cam2} - r_{1x}^{cam2}, r_{2y}^{cam2} - r_{1y}^{cam2}, r_{2z}^{cam2} - r_{1z}^{cam2}), \end{aligned} \tag{16}$$

where l_1 and l_2 are (yet unknown) coefficients. The sought fish coordinates in 3D space are defined by the intersection point of lines L_1 and L_2. However, due to the inevitable errors in reading point coordinates, it is unlikely that the lines would actually intersect. We thus find the sought fish coordinates as a middle point of a vector $\overrightarrow{CD}$, defining the shortest distance between L_1 and L_2. Following conditions apply: $C \in L_1$, $D \in L_2$, and $\overrightarrow{CD} \perp \overrightarrow{v_1}$, and $\overrightarrow{CD} \perp \overrightarrow{v_2}$. The first two conditions imply:

$$C = \begin{cases} C_x = r^1_{1x} + l_1 \left(r^1_{2x} - r^1_{1x}\right) \\ C_y = r^1_{1y} + l_1 \left(r^1_{2y} - r^1_{1y}\right) \\ C_z = r^1_{1z} + l_1 \left(r^1_{2z} - r^1_{1z}\right) \end{cases} \tag{17}$$

and

$$D = \begin{cases} D_x = r^2_{1x} + l_2 \left(r^2_{2x} - r^2_{1x}\right) \\ D_y = r^2_{1y} + l_2 \left(r^2_{2y} - r^2_{1y}\right) \\ D_z = r^2_{1z} + l_2 \left(r^2_{2z} - r^2_{1z}\right) \end{cases} \tag{18}$$

Hence:

$$\overrightarrow{CD} = \begin{cases} \left(r^2_{1x} + l_2 \left(r^2_{2x} - r^2_{1x}\right)\right) - \left(r^1_{1x} + l_1 \left(r^1_{2x} - r^1_{1x}\right)\right) \\ \left(r^2_{1y} + l_2 \left(r^2_{2y} - r^2_{1y}\right)\right) - \left(r^1_{1y} + l_1 \left(r^1_{2y} - r^1_{1y}\right)\right) \\ \left(r^2_{1z} + l_2 \left(r^2_{2z} - r1z^2\right)\right) - \left(r^1_{1z} + l_1 \left(r^1_{2z} - r^1_{1z}\right)\right) \end{cases} \tag{19}$$

The remaining two conditions imply $\overrightarrow{CD} \cdot \overrightarrow{v_1} = \overrightarrow{CD} \cdot \overrightarrow{v_2} = 0$. This can be expressed as:

$$(D_x - C_x) \cdot \left(r^1_{2x} - r^1_{1x}\right) + (D_y - C_y) \cdot \left(r^1_{2y} - r^1_{1y}\right) + (D_z - C_z) \cdot \left(r^1_{2z} - r^1_{1z}\right) = 0 \tag{20}$$

and:

$$(D_x - C_x) \cdot \left(r^2_{2x} - r^2_{1x}\right) + (D_y - C_y) \cdot \left(r^2_{2y} - r^2_{1y}\right) + (D_z - C_z) \cdot \left(r^2_{2z} - r^2_{1z}\right) = 0 \tag{21}$$

We solve the above equations for l_1 and l_2. To do this, we rewrite them in the following form, using relations (17) and (18):

$$\begin{cases} \alpha_1 l_1 + \beta_1 l_2 + \gamma_1 = 0 \\ \alpha_2 l_1 + \beta_2 l_2 + \gamma_2 = 0 \end{cases} \tag{22}$$

where:

$$\begin{cases} \alpha_1 = -1 \cdot \left(\left(r^1_{2x} - r^1_{1x}\right)^2 + \left(r^1_{2y} - r^1_{1y}\right)^2 + \left(r^1_{2z} - r^1_{1z}\right)^2\right) \\ \beta_1 = \left(r^2_{2x} - r^2_{1x}\right) \cdot \left(r^1_{2x} - r^1_{1x}\right) + \left(r^2_{2y} - r^2_{1y}\right) \cdot \left(r^1_{2y} - r^1_{1y}\right) + \left(r^2_{2z} - r^2_{1z}\right) \cdot \left(r^1_{2z} - r^1_{1z}\right) \\ \gamma_1 = \left(r^2_{1x} - r^1_{1x}\right) \cdot \left(r^1_{2x} - r^1_{1x}\right) + \left(r^2_{1y} - r^1_{1y}\right) \cdot \left(r^1_{2y} - r^1_{1y}\right) + \left(r^2_{1z} - r^1_{1z}\right) \cdot \left(r^1_{2z} - r^1_{1z}\right) \end{cases} \tag{23}$$

and:

$$\begin{cases} \alpha_2 = -1 \cdot \left(\left(r^1_{2x} - r^1_{1x}\right) \cdot \left(r^2_{2x} - r^2_{1x}\right) + \left(r^1_{2y} - r^1_{1y}\right) \cdot \left(r^2_{2y} - r^2_{1y}\right) + \left(r^1_{2z} - r^1_{1z}\right) \cdot \left(r^2_{2z} - r^2_{1z}\right)\right) \\ \beta_2 = \left(r^2_{2x} - r^2_{1x}\right)^2 + \left(r^2_{2y} - r^2_{1y}\right)^2 + \left(r^2_{2z} - r^2_{1z}\right)^2 \\ \gamma_2 = \left(r^2_{1x} - r^1_{1x}\right) \cdot \left(r^2_{2x} - r^2_{1x}\right) + \left(r^2_{1y} - r^1_{1y}\right) \cdot \left(r^2_{2y} - r^2_{1y}\right) + \left(r^2_{1z} - r^1_{1z}\right) \cdot \left(r^2_{2z} - r^2_{1z}\right) \end{cases} \tag{24}$$

This yields:

$$l_1 = \frac{\gamma_1 - \frac{\beta_1}{\beta_2}\gamma_2}{\alpha_1 - \frac{\alpha_2\beta_1}{\beta_2}} \tag{25}$$

and:

$$l_2 = \frac{\gamma_2 - \alpha_2 l_1}{\beta_2} \tag{26}$$

Eventually, we substitute (25) and (26) into (17) and (18) to find the desired coordinates of the approximated location of the triangulated point of interest (fish coordinates) in 3D space:

$$P = \begin{cases} x = \frac{C_x + D_x}{2} \\ y = \frac{C_y + D_y}{2} \\ z = \frac{C_z + D_z}{2} \end{cases} \tag{27}$$
